# Convergent genomic and pharmacological evidence of PI3K/GSK3 signaling alterations in neurons from schizophrenia patients

**DOI:** 10.1038/s41386-020-00924-0

**Published:** 2020-12-07

**Authors:** Laura Stertz, Jessica Di Re, Guangsheng Pei, Gabriel R. Fries, Emily Mendez, Shenglan Li, Laura Smith-Callahan, Henriette Raventos, Jerricho Tipo, Rohan Cherukuru, Zhongming Zhao, Ying Liu, Peilin Jia, Fernanda Laezza, Consuelo Walss-Bass

**Affiliations:** 1 Louis A. Faillace, MD, Department of Psychiatry and Behavioral Sciences, McGovern Medical School, University of Texas Health Science Center at Houston, Houston, TX USA; 2 Department of Pharmacology and Toxicology, University of Texas Medical Branch, Galveston, TX USA; 3 Center for Precision Health, School of Biomedical Informatics, University of Texas Health Science Center at Houston, Houston, TX USA; 4 Institute of Molecular Medicine for the Prevention of Human Diseases, McGovern Medical School, University of Texas Health Science Center at Houston, Houston, TX USA; 5 Centro de Investigacion en Biologia Celular y Molecular, Universidad de Costa Rica, San Jose, Costa Rica; 6 School of Medicine, University of Texas Medical Branch, Galveston, TX USA

**Keywords:** Cellular neuroscience, Gene expression analysis

## Abstract

Human-induced pluripotent stem cells (hiPSCs) allow for the establishment of brain cellular models of psychiatric disorders that account for a patient’s genetic background. Here, we conducted an RNA-sequencing profiling study of hiPSC-derived cell lines from schizophrenia (SCZ) subjects, most of which are from a multiplex family, from the population isolate of the Central Valley of Costa Rica. hiPSCs, neural precursor cells, and cortical neurons derived from six healthy controls and seven SCZ subjects were generated using standard methodology. Transcriptome from these cells was obtained using Illumina HiSeq 2500, and differential expression analyses were performed using DESeq2 (|fold change|>1.5 and false discovery rate < 0.3), in patients compared to controls. We identified 454 differentially expressed genes in hiPSC-derived neurons, enriched in pathways including phosphoinositide 3-kinase/glycogen synthase kinase 3 (PI3K/GSK3) signaling, with serum-glucocorticoid kinase 1 (SGK1), an inhibitor of glycogen synthase kinase 3β, as part of this pathway. We further found that pharmacological inhibition of downstream effectors of the PI3K/GSK3 pathway, SGK1 and GSK3, induced alterations in levels of neurite markers βIII tubulin and fibroblast growth factor 12, with differential effects in patients compared to controls. While demonstrating the utility of hiPSCs derived from multiplex families to identify significant cell-specific gene network alterations in SCZ, these studies support a role for disruption of PI3K/GSK3 signaling as a risk factor for SCZ.

## Introduction

Human-induced pluripotent stem cell (hiPSC) technology, and further differentiation into specific brain cell types, is an emerging approach to study cellular models of schizophrenia (SCZ) and other psychiatric disorders. The functional significance of potential SCZ susceptibility loci [1] can be assessed using hiPSC technology [2, 3]. However, current high costs and technical burdens have restricted hiPSC studies to small sample size, limiting the ability to identify significant disease-relevant genomic signals. A transcriptome study utilizing neurons derived from a childhood-onset SCZ cohort identified overlap of neuronal transcriptional signatures with postmortem adult brains but failed to identify definitive differentially expressed genes (DEGs) [4]. The authors concluded that they were underpowered to detect such differences.

Studies of cell lines derived from families with multiple affected individuals, in particular families from genetically homogeneous populations, may provide an important strategy to overcome the dilution of genetic effects typical of case-control studies, as persons with genetic diseases in these populations are more likely to share the same genetic mutations, and thus molecular and cellular profiles, than persons from genetically diverse populations, thus facilitating identification of causative gene networks [5]. Based on this, we hypothesized that generation of hiPSCs from subjects carrying common genetic alterations would empower the identification of global transcriptome alterations and gene network pathways in a cell-type- and disease-specific way.

The population of the Central Valley of Costa Rica (CVCR) is considered a genetically homogeneous population. It is well known in studies of complex illnesses due to its centralization of health care, large family sizes, and high rate of compliance of patients. For 200 years this population grew in relative isolation, ideal for studying the genetic factors in complex illnesses [6]. We have performed in-depth genetic studies of SCZ families from the CVCR and have identified several potential SCZ candidate genes [7–15].

We generated hiPSC-derived neuronal precursor cells (NPCs) from ten individuals from a multiplex CVCR SCZ family and from three unrelated CVCR individuals, and further differentiated these cells into neurons, for a transcriptome analysis based on the SCZ phenotype. We identified 454 DEGs in hiPSC–neurons in patients compared to controls, and the phosphoinositide 3-kinase/glycogen synthase kinase 3 (PI3K/GSK3) signaling pathway was among the most significant gene networks identified by enrichment analysis. Among the DEGs in the PI3K/GSK3 signaling pathway was serum-glucocorticoid kinase 1 (SGK1), an inhibitor of glycogen synthase kinase 3β (GSK3β) [16–18], which has been implicated in SCZ and in neuronal morphogenesis [19, 20]. Based on this, we assessed the effect of inhibitors of SGK1 and GSK3, downstream effectors of PI3K, in hiPSC–neurons, and found differential sensitivity to these inhibitors in patients compared to controls. These findings support the notion that alterations in PI3K/GSK3 signaling pathways may represent a mechanism of risk for SCZ.

## Materials and methods

### Sample characteristics

**Fig. 1:**
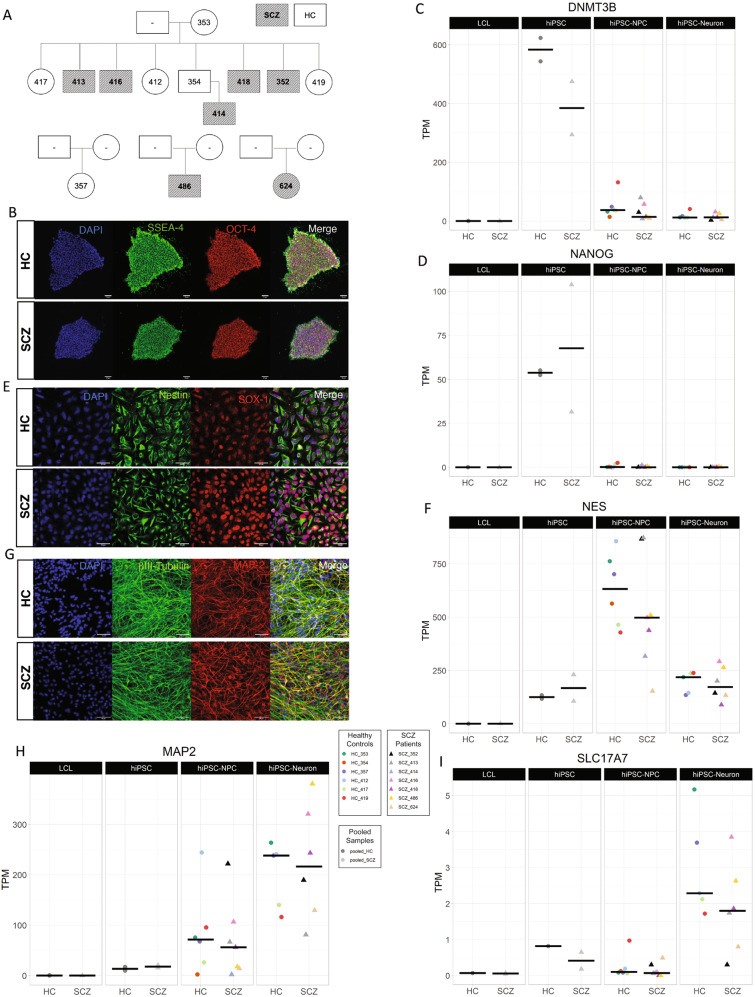
Generation and neural differentiation of hiPSCs. **A** Family pedigrees from the Central Valley of Costa Rica. Individuals from four different families were included in this study. Squares represent males, and circles represent females. Subjects with a number identification represent those included in this study from whom hiPSC, NPC, and neuronal cell lines were made. In total, 39 cell lines were derived from 13 subjects. Subjects in shaded boxes were diagnosed as having schizophrenia (SCZ), subjects in white boxes represent healthy controls (HC). **B** Representative images of hiPSCs derived from SCZ patients and HC expressing pluripotent markers SSEA4 (green) and Oct4 (red). Gene expression (TPM) levels of pluripotency cell markers **C** DNMT3B and **D** NANOG is increased in hiPSCs when compared to LCLs, hiPSC–NPCs, and hiPSC–neurons. **E** Representative images of hiPSC–NPC positive for Nestin (green) and SOX-1 (red). **F** hiPSC–NPCs have higher expression levels of NPC cell marker Nestin (NES), with no significant differences between patients and controls. **G** Representative images of 3-week-old hiPSC–neurons positive for βIII tubulin (green) and MAP2 (red). The hiPSC–neurons have higher expression levels of **H** MAP2 and **I** SLC17A7 (vGlut1), with no significant differences between SCZ and HC. Comparison between groups: Mann–Whitney. No statistical test was performed on the pooled samples. Gene expression levels are expressed in TPM (Transcripts Per Kilobase Million). Line on plots represents median.

De-identified cell lines were generated from a CVCR SCZ multiplex family comprised of ten individuals, four females and six males, of which four male siblings are affected with SCZ, and all females are unaffected. Cell lines from three additional unrelated CVCR individuals (two SCZ affected, one male and one female, and one unaffected female) were also generated (Fig. 1A). All subjects were carefully characterized in previous studies, according with the Principles of the Declaration of Helsinki, and lymphoblastoid cell lines (LCLs) were already generated from each subject, as previously described [8].

### Reprogramming of human LCLs with episomal vectors

LCLs (*n* = 13) were reprogrammed into hiPSCs using the Epi5™ Episomal iPSC reprogramming Kit (Thermo Fisher Scientific, Waltham, MA), containing Oct4, Sox2, Klf4, L-Myc, and Lin28. Nucleofection was conducted using the Cell Line Optimization Nucleofector™ X Kit for the 4D-Nucleofector™ System (program EW113, Lonza, Basal, Switzerland). Briefly, 2 × 10^6^ cells from each subject were nucleofected with Epi5™, stabilized in RPMI1640 medium with 10% FBS (Gibco) and 1x Penicillin Streptomycin (PenStrep, Gibco-Thermo Fisher Scientific, Waltham, MA) for 3 days, and then transferred to 60 mm dishes coated with Membrane Matrix (Corning Matrigel hESC-Qualified Matrix (Corning) or Geltrex™ LDEV-Free Reduced Growth Factor Basement Membrane Matrix (Gibco-Thermo Fisher Scientific, Waltham, MA)). RPMI medium was then replaced by TeSR™-E8™ (StemCell Technologies, Vancouver, Canada) and 1x PenStrep (Gibco) every day until the appearance of colonies (15–30 days), at which time clones were manually chosen and expanded. iPSC clones from each of the cell lines were stained for the pluripotency markers SSEA4 and Oct4, and karyotyping analysis by standard G-banding technique was carried out by KaryoLogic, Inc. (Research Triangle Park, NC, USA). Details regarding clinical information for each donor and cell lines used for experiments described below are found in Supplementary Table S1.

### Neuronal precursor cells (NPC) differentiation

One clone from each subject (*n* = 13) was differentiated into hiPSC–NPC using AggreWell™ methodology (StemCell Technologies), that involves the formation of embryoid bodies (EB) using STEMdiff™ Neural Induction Medium, according to manufacture instructions. After 5 days, EBs were plated in six-well plates coated with Membrane Matrix. Rosette selection was performed after 7 days using STEMdiff Rosette Selection Reagent (StemCell Technologies) and plated on Membrane Matrix-coated wells. STEMdiff™ Neural Induction Medium was changed every day. When confluent, hiPSC–NPC were lifted using Accutase (Innovative Cell Technologies, Inc.) and expanded on Membrane Matrix-coated plates in Neurobasal medium 50/50 (50% DMEM/F12 (Corning), 50% Neurobasal Medium (Gibco), 1x GlutaMAX (Gibco), 1x NEAA (Gibco), 1x PenStrep (Gibco), 1x N-2 Supplement (Gibco), 1x B27 minus vitamin A (Gibco)) supplemented with 20 ng/ml FGF-Basic (AA 1–155) Recombinant Human Protein (Gibco) until passage 3. hiPSC–NPCs were immunocytochemically characterized using markers for proteins highly expressed by NPCs (Nestin and SOX-1).

### Cortical neuron differentiation

Differentiation of hiPSC–NPCs into cortical neurons (hiPSC–neuron) was performed as previously described [21], with a few modifications. Briefly, NPCs were cultivated in pre-coated Laminin/Poly-L-Ornithine plates in Neurobasal Medium 50/50 supplemented with 20 ng/ml BDNF (PeproTech), 20 ng/ml GDNF (PeproTech), 1 mM dibutyryl-cyclic AMP (Sigma), 200 nM ascorbic acid (Sigma), 10 ng/mL IGF-1 (PeproTech), and 10 ng/ml WNT-3A (R&D). hiPSC–neurons were stained for βIII tubulin, MAP2, and NeuN after 3 weeks of differentiation. Images obtained by Leica TCS SP8 Confocal System were analyzed using ImageJ (NIH). Total nuclei in processed images were counted using CellProfiler IdentifyPrimaryObjects function [22], and the percent of nuclei positive for the target of interest (NeuN, MAP2, and βIII tubulin) was counted using ImageJ. See Supplementary Methods for immunocytochemistry details.

### RNA sequencing

Cells from each cell type/subject, matched for passage and mycoplasma-free, were cultured in triplicate, and replicates were combined for RNA extraction. Due to budget limitations, for the LCLs and hiPSCs only, subjects were pooled into four groups based on phenotype, resulting in two unaffected and two affected groups for each cell type. In total, RNA from 32 distinct samples (LCLs (*n* = 4), hiPSCs (*n* = 4), hiPSC–NPCs (*n* = 13), and hiPSC–neurons (*n* = 13)) was sequenced. Total RNA was extracted and purified using the RNeasy Plus Mini kit (Qiagen, Hilden Germany). Quality and integrity were assessed by the Agilent Bioanalyzer 2100 system (Agilent Technologies, Santa Clara, CA) and agarose gel electrophoresis. A total of 1 μg RNA per sample was used for mRNA-seq library construction using NEBNext® Ultra™ RNA Library Prep Kit for Illumina® (Illumina Inc., San Diego, CA). Paired-end sequencing reads (150 bp) were generated on an Illumina HiSeq2000 platform (Q30 > 80%) (Illumina) at Novogene Bioinformatics Institute (Chula Vista, CA).

### RNA data processing

Raw mRNA sequence reads were pre-processed using cutadapt (v. 1.15) to remove bases with quality scores < 20 and adapter sequences [23], followed by alignment of clean RNA-seq reads to GRCh38.83 with STAR (v2.5.3a) [24]. Uniquely mapped reads overlapping genes were counted by htseq-count with default parameter using annotation from ENSEMBL v83. Only genes with >5 reads in at least one sample were retained. Reads counts were normalized to the aligned Reads Per Kilobase Million to obtain the relative expression levels.

### RNA deconvolution analysis

Cell type and developmental stage composition analysis was performed using a recent method [25]. Briefly, we adapted regression calibration matrices, originally created from human single-cell and brain homogenate RNA-seq data sets [26–30], using methods previously established for deconvolution of epigenetic data [31, 32]. We then projected normal-transformed TPM of barcode genes for each sample into the design matrix with the “minfi” Bioconductor “projectCellType()” function [33], and calculated RNA fractions by normalizing the fitted model scores to the total scores for each line for all subtypes. Fetal developmental stage ratios were summed after fitting to create a single fetal ratio score for statistical testing and visualization (NCX_Fetal). Thus, this analysis subsets fractions of our data into RNA fractions from five different human ectoderm-derived cell types (NPC, neurons, astrocytes, OPCs, and oligodendrocytes) plus iPSCs, in six different human neocortical (NCX) developmental stages (iPSC, Fetal, Infant, Child, Teens, and Adult). Visualization of cell-type ratios and statistical comparisons was performed in R, and cell-type and maturity fractions were compared between groups using two-sample *t*-tests with Holm–Sidak correction unless otherwise noted.

### Differential expression analysis

We excluded Y chromosome genes to avoid bias effects due to unbalanced sex of samples. Retained genes (raw reads count) were submitted for differential expression analysis of cases compared to controls in each cell type with DESeq2 software [34], which implements a model based on the negative binomial distribution. Resulting *p* values were adjusted using the Benjamini and Hochberg’s (BH) approach [35] to control for false discovery rate (FDR). Genes with fold change (FC) > 1.5 and FDR < 0.3 were considered to be significant. Pathway analysis [36–38], principal components analysis (PCA), and linear mixed model analysis [39] were performed as described in Supplementary Methods.

### Enrichment analysis using publicly available data sets

We assessed enrichment of genome-wide association study (GWAS) signals using Multi-markers Analysis of GenoMic Annotation (Supplementary Methods) and summary statistics for SCZ [1], bipolar disorder (BD) [40], SCZ + BD [41], OCD [42], suicide attempt in SCZ [43], PTSD [44], major depressive disorder [45], autism spectrum disorder [46], and ADHD [47]. We also assessed concordance of our gene set with gene expression data sets from human postmortem brain and hiPSC studies [4, 48–50].

### Inhibition of SGK1 and GSK3 and neurite imaging

Neuronal cell lines from six siblings in the multiplex family (three controls and three patients) were chosen to achieve the most homogenous genetic background possible for functional studies. NPC cell lines from these subjects, derived from the same iPSC clones used for the RNA-seq studies, were differentiated into neurons and at day 21 were treated with 0.5% DMSO (control), 20 μm GSK3 inhibitor CHIR99021 (Tocris), 20 μm SGK1 inhibitor GSK650394 (Selleck) for 24 h, or 20 μm CHIR99021 for 14 h followed by 10 h with 20 μm GSK650394. Details of the imaging protocol are described in Supplementary Methods. Average fluorescent intensity was calculated by ImageJ for both βIII tubulin and fibroblast growth factor 12 (FGF12) and analyzed to ensure that each measurement was independent of potential changes in the number or size of βIII tubulin positive neurites.

### Reverse transcription quantitative polymerase chain reaction

RT-qPCR was performed to assess expression of SGK1 in neurons from the same six siblings used for the pharmacological studies (Supplementary Methods).

## Results

### Cell line characterization

Karyotype analysis of hiPSCs revealed two patients and two controls to have 5% chromosome 1 aneuploidy. All other cell lines were normal (Table S1). Studies have reported widespread somatic mosaicism in the human body, suggesting reprogramming does not necessarily lead to de novo CNVs in iPSC [51]. Nevertheless, as the aneuploidy is found in equal number of patients and controls in a very small percentage of cells, we do not expect this to influence the study. hiPSC characterization and differentiation to hiPSC–NPC and hiPSC–neurons were performed for each subject (Fig. 1 and Supplementary Fig. S1). Neurons exhibited higher expression levels of neuronal markers MAP2 and SLC17A7 (vGlut1) compared to LCLs, hiPSC, and hiPSC–NPCs (Fig. 1H, I). No differences were found between HC and SCZ cell lines on the expression of NPC and neuronal markers.

### RNA sequencing

RNA-seq data were generated from a total of 32 samples (LCLs (*n* = 4), hiPSCs (*n* = 4), hiPSC–NPCs (*n* = 13), and hiPSC–neurons (*n* = 11)), representing four unique cell lines from 13 individuals. Two NPC cell lines did not differentiate into viable neurons (354 and 414) and were thus excluded from the hiPSC–neuron analysis. In all, 2.25 billion paired-end reads were obtained, and the median number of uniquely mapped read pairs per sample was 24.98 million at a 90.4–95.8% ratio, indicating a very small fraction of rRNA reads. A total of 30,500 genes (based on ENSEMBL v83 annotations) were expressed at a level deemed sufficient for analysis. Among them, 17,351 were protein coding genes, 3211 were lincRNAs, and the remaining were of various biotypes (Supplementary Fig. S2). No expression of episomal vector genes was observed.

### Sex validation and observation for contamination or aberrant X-inactivation

Using XIST on chrX and the expression of six genes on chrY (USP9Y, UTY, NLGN4Y, ZFY, RPS4Y1, and TXLNGY), we confirmed that all samples show a correct expression pattern according to their gender (Supplementary Fig. S3). No other indication of contamination or aberrant X-inactivation was detected.

### Cell type and developmental stage composition analysis

**Fig. 2:**
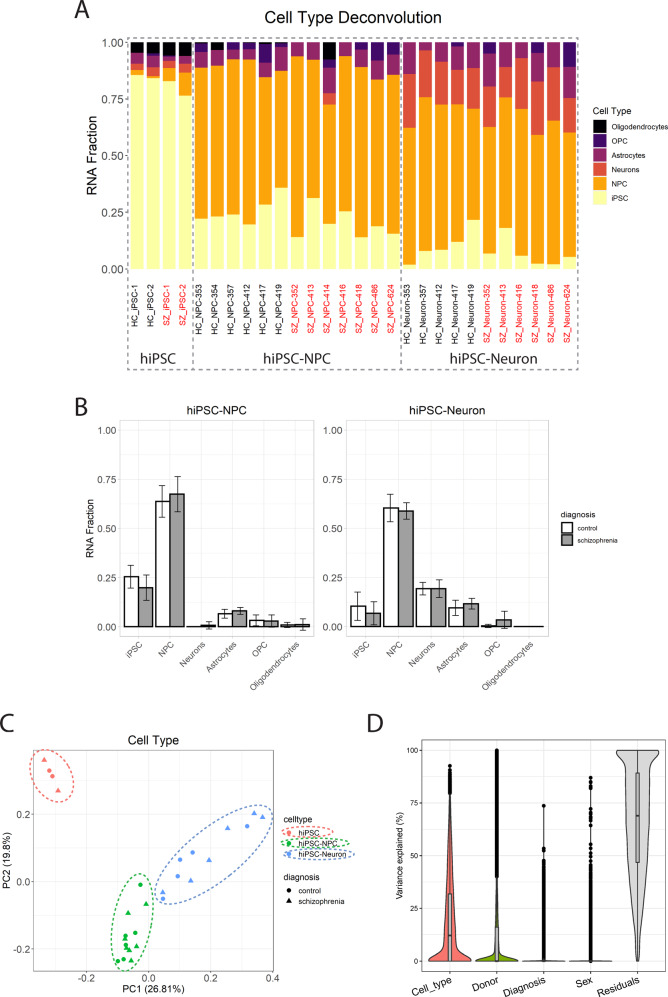
Gene expression is influenced by cell-type composition. **A** Fraction of relative cell-type RNA composition is determined by the cell-type regression calibration deconvolution model. Horizontal axis labels SCZ (red) and control (black) NPC and neuron cell lines, as well as pooled iPSC lines. **B** No significant differences in cell composition were observed in hiPSC–NPCs and hiPSC–neurons between cases and controls. **C** Principal components analysis (PCA) of gene expression data (scaled TPM) from hiPSCs (red), hiPSC–NPC (green), and hiPSC–neurons (blue) shows that both control (circles) and SCZ (triangles) samples cluster by cell type. **D** Variance partition analysis with violin plots of the percentage of variance explained by each variable over all the genes.

Given that bulk RNA-seq analysis can reflect multiple constituent cell types across different developmental stages, we performed deconvolution analysis to calculate cell-type RNA fraction scores and NCX human developmental stage for each of our cell lines. We found that as hiPSCs differentiated into NPCs and neurons, the iPSC fraction progressively decreased while the more mature neuron RNA fraction increased (Fig. 2A and Supplementary Fig. S4A). Specifically, hiPSC–neurons had a lower iPSC RNA fraction than hiPSC–NPCs and a higher neuron RNA fraction than hiPSC–NPCs. There was no significant difference in any cell-type RNA fraction between SCZ and HC (Fig. 2B). Developmental stage deconvolution also revealed a rise in RNA fractions representing the fetal and adult stages over differentiation (NCX_Fetal and NCX_Adult, Supplementary Fig. S4).

PCA of the expression data illustrates that hiPSCs, hiPSC–NPCs, and hiPSC–neurons separate along the first principal component (PC), which explains 26.81% of the variance (Fig. 2C).

### Differential expression analysis

**Fig. 3:**
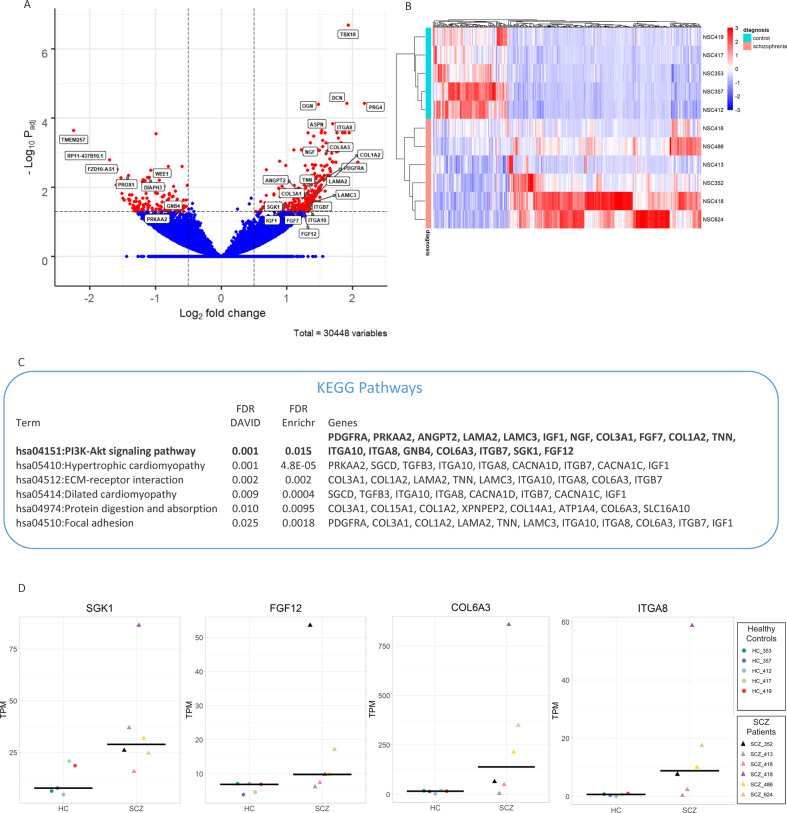
Differential expression in neurons of schizophrenia patients compared to controls. **A** Volcano plot showing log2 fold change between cases and controls. Genes are colored based on significance: red (454 differentially expressed genes (DEGs), 332 upregulated and 122 downregulated) and blue (not significant) with cutoffs of log2 FC > 0.5 or <−0.5 and BH FDR corrected *p* value < 0.05. **B** Heatmap of DEGs by sample. **C** Enrichment pathway analysis of DEGs using DAVID and Webgestalt (demonstrated by enrichment ratio) implicated a set of KEGG pathways related to the PI3K/GSK3 signaling pathway and extracellular matrix organization. **D** Gene expression of selected genes from the PI3K/GSK3 signaling pathway in hiPSC–neurons.

We compared cases against controls in NPCs and neurons separately. Analyses identified two DEGs (TXLNG and AP1S*2*) in NPCs and 454 in neurons (Fig. 3 and Supplementary Table S2). Variance partition analysis showed that cell type had the largest effect on expression and explained a median of 12% of the expression variation (Fig. 2D). Of interest, among the DEGs identified in neurons were CACNA1C, C4A, and ZNF804A, previously identified as SCZ candidate genes by GWAS [1, 52, 53]. Pathway analysis of the neuronal DEGs, using DAVID, EnrichR, and Webgestalt, all implicated a coherent set of KEGG networks related to the PI3K/GSK3 signaling pathway and to extracellular matrix (ECM) organization, significant after FDR correction (Fig. 3C), in which many of the same genes found in ECM pathways were found in the PI3K/GSK3 pathway. Among the genes identified as part of the PI3K/GSK3 pathway were SGK1, an inhibitor of GSK3β [16–18], overexpressed in patients. Also identified in this pathway were FGF12, a primary constituent of the voltage-gated Na^+^ (Nav1.2) channel in the brain [54], and several ECM genes known to function via PI3K/GSK3 signaling, including ITGA8 [55] and collagen subunits [56, 57] (Fig. 3D).

### Concordance with genetic/genomic studies

While we did not find significant enrichment for GWAS signals in our DEGs (SCZ—*p* = 0.349, BD + SCZ—*p* = 0.427, ASD—*p* = 0.435, MDD—*p* = 0.572, PTSD—*p* = 0.572, suicide attempt in SCZ—*p* = 0.739, OCD—*p* = 0.74, BD—*p* = 0.954, ADHD—*p* = 0.966), we found overlap of five DEGs (CACNA1C, C4A, GRAMD1B, PLCL1, and ZNF804A) with genes annotated to genome-wide significant SNPs in SCZ [1].

We evaluated concordance of our findings with two SCZ gene expression studies in the dorsolateral prefrontal cortex [48, 49]. We identified 20 and 6 concordant genes, respectively, in each comparison (Supplementary Table S3). CREBRF was identified in all three studies, with the same direction of change (increase in patients). We also assessed concordance of our gene set with that from SCZ hiPSC-derived neural cells [4, 50]. Of the 454 DEGs in our study, 323 were measured by Hoffman et al. [4], but there was no significant correlation between changes. Evgrafov et al. [50] reported a negative correlation for all genes in their data set and that of the Hoffman et al. [4] study. We found two genes in common with the study by Evgrafov et al., IGFBP5 and AHGAP26, with the same direction of change. Also, Evgrafov et al. identified WNT5A as one of their top DEGs, whereas we identified WNT5B, with the same direction of change. Both WNT genes are members of the canonical Wnt signaling pathway, which impinges on GSK3β.

### Effect of SGK1 and GSK3 inhibition on βIII tubulin and FGF12 levels in neurites

To establish a functional role for the PI3K/GSK3 pathway in SCZ, and specifically for SGK1 as part of this pathway, we assessed the effect of pharmacological inhibitors of SGK1 and GSK3 on levels of FGF12 and the cytoskeleton marker βIII tubulin in neurites. We chose to measure βIII tubulin because GSK3 plays a key role in neurite formation by directly influencing tubulin stability [20], and specifically modulates βIII tubulin expression [58], a beta tubulin isoform selectively affected during neurite formation [59]. FGF12 was chosen as an outcome marker because of the suggestive transcriptome evidence of its involvement in the PI3K/GSK3 pathway, and our previous studies showing GSK3 as a regulator of intracellular FGFs [60]. Furthermore, FGF12 is involved in neurite formation [61], a process known to be altered in SCZ [62], and has high similarity in sequence and functionality to FGF14, which has been implicated in SCZ [63].

**Fig. 4:**
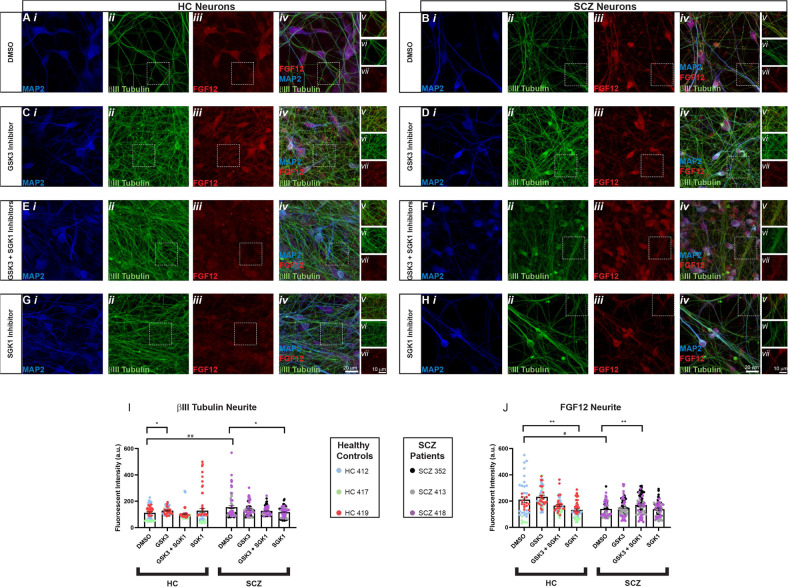
βIII tubulin and FGF12 are differentially regulated in neurites of HC and SCZ by PI3K/GSK3 inhibitors. **A**–**I** Representative images of neurons from healthy controls (HC) and schizophrenia (SCZ) cell lines. i MAP2 (blue), ii β-III tubulin (green), iii FGF12 (red), and iv merge of all three channels with v zoom to ROI used for analysis of vi βIII tubulin and vii FGF12. Squares in panels (ii–iv) represent the ROI used for analysis based on the βIII tubulin fluorescent intensity threshold. **A** HC neurons treated for 24 h with DMSO control. **B** SCZ neurons treated for 24 h with DMSO control. **C** HC neurons treated for 24 h with GSK3 inhibitor CHIR99021. **D** SCZ neurons treated for 24 h with GSK3 inhibitor CHIR99021. **E** HC neurons treated for 14 h with GSK3 inhibitor CHIR99021 followed by 10-h treatment with SGK1 inhibitor GSK650394. **F** SCZ neurons treated for 14 h with GSK3 inhibitor CHIR99021 followed by 10-h treatment with SGK1 inhibitor GSK650394. **G** HC neurons treated for 24 h with SGK1 inhibitor GSK650394. **H** SCZ neurons treated for 24 h with SGK1 inhibitor GSK650394. Scale bars represent iv 20 μm or vii 10 μm. **I** βIII immunofluorescence in neurites is increased in SCZ patients compared to healthy controls when treated for 24 h with DMSO (two-way mixed model ANOVA with Sidak’s multiple comparison’s test, *p* = 0.0095). Twenty-four-hour treatment with 20 μm GSK3 inhibitor CHIR99021 increases fluorescent intensity in HC neurons, but not SCZ neurons, while 24 h with SGK1 inhibitor GSK650394 decreases fluorescent intensity in SCZ neurons, but not healthy controls (two-way mixed model ANOVA with Dunnett’s multiple comparison’s test, *p* = 0.0215 (HC GSK3) and *p* = 0.0124 (SCZ SGK1)). **J** FGF12 immunofluorescence in neurites is decreased in SCZ neurons compared to healthy controls when treated for 24 h with DMSO (two-way mixed model ANOVA with Sidak’s multiple comparison’s test, *p* = 0.0107). Treatment with SGK1 inhibitor GSK650394 decreases fluorescent intensity in HC neurons, while 14-h treatment with GSK3 inhibitor CHIR99021 followed by 10-h treatment with SGK1 inhibitor GSK650394 increased fluorescent intensity of FGF12 in SCZ neurons (two-way mixed model ANOVA with Dunnett’s multiple comparison’s test, *p* = 0.0009 (HC SGK1) and *p* = 0.0073 (SCZ GSK3 + SGK1)). Data are mean ± SEM. ^#^ indicates *p* < 0.05 and ^##^ indicates *p* < 0.01 by two-way ANOVA with Sidak’s multiple comparison’s test. * indicates *p* < 0.05 and ** indicates *p* < 0.01 by two-way ANOVA with Dunnett’s multiple comparison’s test. Dots represent measurements from single neurons.

We found a significant correlation between RNA-seq and qPCR expression data for SGK1 in the hiPSC–neurons (*r* = 0.9751) (Supplementary Fig. S5). No differences between HC and SCZ were found in the baseline characterization of these cell lines using NeuN, MAP2, and βIII tubulin (Supplementary Fig. S5). Around 40% of cells were positive for MAP2, indicating a mixture of mature and immature neuronal cells in culture. We found significant differences in sensitivity to inhibitors in patients compared to controls. Patients treated with DMSO (vehicle) had increased βIII tubulin levels in neurites compared to controls (Fig. 4Avi, Bvi, I). SGK1 inhibition (which effectively abrogates SGK1-induced GSK3 inhibition) caused a decrease in neurite βIII tubulin in patients but not in controls, whereas GSK3 inhibition led to an increase in neurite βIII tubulin levels in controls but not in patients (Fig. 4Cvi, Dvii, Gvi, Hvii, I). Therefore, inhibition of SGK1 in patients led to reciprocal levels of βIII tubulin in controls treated with GSK3 inhibitor. In regards to FGF12, SGK1 inhibition led to decreased levels in control subjects but not in patients (Fig. 4Gvii, Hvii, J). The GSK3 inhibitor CHIR99021 did not cause any effect in either subject group (Fig. 4Cvii, Dvii, J). Overall, these results suggest differential sensitivity to inhibition of PI3K/GSK3 signaling in patients compared to controls, perhaps due to the overexpression of SGK1 that is found in these patients.

## Discussion

By generating hiPSCs derived from individuals from a genetically homogenous population, we detected significant gene expression alterations in pathways that may modulate SCZ risk. Overall, we found a stronger effect in hiPSC–neurons, compared to NPCs, consistent with the hypothesis that neurons are the cell type most relevant to SCZ risk [64].

Initial RNA-seq analyses validated the sex of each cell line and excluded the possibility of sample cross-contamination or aberrant X-inactivation exclusion. It is not uncommon for such errors to be introduced during this protocol [65], and only a few studies have reported such an in-depth validation analysis.

We found concordance of some of our DEGs with those identified in other SCZ genetic/transcriptomic studies, including differential expression of GWAS- associated genes CACNA1AC, C4A, GRAMD1B, PLCL1, and ZNF804A [1]. To our knowledge, this is the first report of altered expression of these genes in SCZ-derived neurons.

Neuronal DEGs were enriched in pathways related to PI3K/GSK3 signaling and ECM organization, supporting impairments in these processes in SCZ [66]. Among the top 5 DEGs were DCN, PRG4, OGN, ITGA8, and ASPN, all of which are involved in mediating cell–cell interactions, ECM formation, and cell migration. Importantly, ECM proteins are known to function via PI3K/GSK3 signaling, including ITGA8 [55], DCN [67, 68], and OGN [69], identified as top DEG’s, and ITGA8 and collagen subunits [56, 57] identified as part of the PI3K/GSK3 pathway.

Pathway analysis is an agnostic way of grouping genes together based on literature and known gene functions, and the interpretation of these analyses should be guided by relevance to the disease of interest [70]. The PI3K/GSK3 signaling pathway was among the most significant canonical pathways identified by enrichment analysis in our study. This pathway, including GSK3, has been strongly implicated in the pathophysiology of SCZ and as a pathological mediator of genetic and environmental programming during development [19, 66]. Among the genes associated with this pathway we found SGK1, a downstream effector of PI3K that inhibits GSK3 activity in an AKT-independent manner [16, 17]. Based on the identification of SGK1 as a novel potential mediator of SCZ risk as part of the PI3K/GSK3 pathway, we performed pharmacological studies to validate its role in our cellular model. We found decreased sensitivity to PI3K/GSK3 signaling inhibition in SCZ patients. Specifically, the findings that pharmacological inhibition of GSK3 with CHIR99021, which acts similarly to SGK1-induced GSK3 inactivation by phosphorylation, did not alter FGF12 or βIII tubulin expression in SCZ neurons points toward an already saturated inhibition of GSK3, perhaps due to the increased expression of SGK1 in these SCZ subjects.

GSK3 is a downstream effector of several signaling pathways, including the PI3K pathway, the mTOR pathway, and the Wnt canonical pathway, suggesting that GSK3 may function to coordinate and integrate signaling within these pathways [20]. SCZ is a complex psychiatric disorder in which different affected individuals may carry causative variants in any of a wide number of genes which impinge on common pathways, such as PI3K/GSK3 signaling. In this regard, a recent transcriptomic study of SCZ neural progenitor cells identified the Wnt signaling pathway as the most enriched [50], with WNT5A as one of the top DEGs, whereas we identified WNT5B, with the same direction of change. Taken together with our functional studies, these findings in SCZ-derived neural cell lines strongly support a role for genes impinging on GSK3 function in risk for SCZ. Because of the genetic complexity underlying SCZ, we cannot determine whether the observed alterations are directly caused by any particular mutation(s) that may be carried by the subjects in our study. Further studies with larger samples sizes are needed to clearly determine which causative mutations may lead to PI3K/GSK3 signaling alterations.

Limitations of our study include the differentiation of single iPSC clones per subject and the relatively small sample size. Our functional studies focused on validation of SGK1 in the PI3K/GSK3 pathway. Future investigation is needed to validate and evaluate the potential role of other genes and pathways identified here. Moreover, our pharmacological experiments were limited to assessing the effects of inhibition of PI3K/GSK3 signaling on FGF12 and βIII tubulin. Additional studies are now warranted to determine the role of PI3K/GSK3 signaling on modulation of other genes identified in this study, and the relevance of these changes on cellular features and functions, including neurite morphology, and calcium and sodium channel activity.

To the best of our knowledge, this study is the first to identify PI3K/GSK3 signaling alterations in SCZ neurons. Overall, our results highlight that transcriptomic alterations in SCZ patients are cell type specific and demonstrate the potential of hiPSCs derived from subjects with a common genetic background to identify gene networks and signaling alterations that may underlie the molecular and cellular mechanisms in SCZ.

## Funding and disclosure

This study was supported by a University of Texas System (UT BRAIN) award (CWB) and a Brain and Behavior Research Foundation (NARSAD) Young Investigator Award (LS). JDR was supported by a National Institutes of Health training grant (T32ES007254). FL was supported by 1R01MH124351. ZZ and PJ were supported by National Institutes of Health grant (R01LM012806). The authors declare no competing interests.

## Supplementary information

Supplemental Material

Demographics and quality control

Differential expression of genes in neurons

Concordance of DEGs with SCZ gene expression studies

Characterization of hiPSCs generated from LCLs

Gene feature statistics

Expression pattern of sex chromosome genes

Cell type composition and maturity deconvolution

Characterization of neurons in functional studies
