# Supplementary material for: Convergent genomic and pharmacological evidence of PI3K/GSK3 signaling alterations in neurons from schizophrenia patients
Source: Neuropsychopharmacology. 2020 Dec 7;46(3):673–82. doi: 10.1038/s41386-020-00924-0 (PMC8027596; doi:10.1038/s41386-020-00924-0)
Supplement: Supplementary file 1 — Supplemental Material [file 41386_2020_924_MOESM1_ESM.docx]

**Table S1. Demographics and quality control**

**Table S2. List of differentially expressed genes**

**Table S3. Concordance of DEGs with SCZ gene expression studies in the dorsolateral prefrontal cortex**

**Figure S1. Characterization of hiPSCs generated from LCLs.** Individual representation of hiPSCs from HC and SCZ subjects positive for pluripotency markers SSEA-4 and Oct-4. Cells present typical morphology, with high nucleus:cytoplasm ratio and formation of compact colonies.

**Figure S2. Gene feature statistics**

**Figure S3. Expression pattern of sex chromosome genes**. Graph represents expression of *XIST* on chrX and the sum expression of six genes on chrY (USP9Y, UTY, NLGN4Y, ZFY, RPS4Y1, TXLNGY).

**Figure S4**. **Cell type composition and maturity deconvolution.** (A) RNA cell type deconvolution tracked over differentiation from hiPSC to NPCs to neurons shows a decline in iPSC RNA fraction (left), a rise and then decline in NPC RNA fraction (middle) and a substantial neuron RNA fraction only detected in fully differentiated neurons (right). Error bars represent standard deviation. (B) Fraction of relative RNA maturity determined by the maturity regression calibration deconvolution model. Horizontal axis labels each SCZ (red) and control (black) NPC and neuron cell line as well as pooled iPSC lines. (C) No significant differences in RNA maturity ratios were observed in hiPSC-NPCs and hiPSC-Neurons between cases and controls. While there was a trend for a decrease in immature iPSC maturity ratios in SCZ cases in hiPSC-NPCs, this did not survive multiple hypothesis correction (t test, p = 0.043, alpha = 0.05, statistical significance determined by Holm-Sidak method) (D) RNA maturity deconvolution tracked over differentiation from hiPSC to neurons shows a decline in immature iPSC RNA fraction (left), and a progressive rise in fetal (middle) and adult (right) RNA fractions.

**Figure S5. Characterization of neurons in functional studies.** (A) Differences between HC and SCZ on SGK1 qPCR expression. (B) There is a positive correlation between RNAseq data (TPM) and qPCR data (RQ) for SGK1 gene expression. Comparison between groups: t-test. Correlation: Pearson r. Data shown as mean with SD. (C) Immunofluorescence staining of hiPSC-Neurons positive for βIII-tubulin and MAP-2. There is no statistically significant difference between HC and SCZ on the percentage of cells that are positive for MAP2, βIII-Tubulin (TUBB3) and NeuN. Comparison between groups: t-test. Lines on plots represent mean.

**Supplemental Methods**

**Immunocytochemistry.**

Cells were fixed with 4% paraformaldehyde (Sigma) for 30 minutes at room temperature (RT), then permeabilized and blocked with 0.1% Triton X-100 (Sigma) and 10% donkey or goat serum (depending on secondary antibody used) in PBS for 1h at RT. Samples were incubated with primary antibodies overnight, washed 3 times with PBS, and then incubated with secondary antibody (Molecular Probes) on block buffer for 2 hours at RT. Nuclei were counterstained with DAPI. Cells were washed and mounted using Fluoromount- G (SouthernBiotech, 0100-01).

List of Antibodies and dilutions.

|  | **Antibody** | **Catalog Number** | **Dilution** |
| --- | --- | --- | --- |
| **Primary** | Ms IgG3 SSEA4 | R&D MAB1435 | 1:50 |
|  | Rb IgG Oct-4 | Thermo Fisher Scientific PA5-27438 | 1:250 |
|  | Ms IgG1 Nestin | Thermo Fisher Scientific MA1-110 | 1:250 |
|  | Rb IgG SOX-1 | Abcam ab109290 | 1:250 |
|  | GP MAP-2 | Synaptic Systems 188004 | 1:1000 |
|  | Rb βIII Tubulin | Abcam ab18207 | 1:1000 |
|  | Ms NeuN Alexa 488 Conjugated | Millipore MAB377X | 1:100 |
|  | Ms IgG1 FGF12 | NeuroMabs 75-135 | 1:200 |
|  | Ck MAP-2 | Invitrogen pa1-10005 | 1:2000 |
|  | Rb βIII Tubulin | Abcam ab18207 | 1:2000 |
|  |  |  |  |
| **Secondary** | Anti-Ms IgG1 Alexa 647 | Life Technologies | 1:200 |
|  | Anti-Rb Alexa 488 | Life Technologies | 1:200 |
|  | Anti-Ck Dylight 405 | Jackson ImmunoResearch Laboratories 103-475-155 | 1:200 |
|  | Anti-Ms IgG1 Alexa 4888 | Molecular Probes A21121 | 1:1000 |
|  | Anti-Rb Alexa 647 | Molecular Probes A21245 | 1:1000 |
|  | Anti-Ms IgG3 Alexa 488 | Molecular Probes A21151 | 1:1000 |
|  | Anti-GP Alexa 647 | Molecular Probes A21450 | 1:1000 |
|  | Anti-Rb Alexa 488 | Molecular Probes A11034 | 1:1000 |

**Pathway analysis.** The ENSEMBL identifiers of differentially expressed genes were converted to Entrez Gene IDs using the org.Hs.eg.db package, and Kyoto Encyclopedia of Genes and Genomes (KEGG) pathway enrichment analyses were performed using DAVID WebService (6.8) [36], EnrichR [37], and Webgestalt (Kegg) [38] . For each analysis, the resulting p-values were adjusted using BH [35].

**Principal components analysis (PCA).** PCA was performed on centered and scaled TPM values for each cell type separately, after removing the effect of cell type composition scores.

**Linear mixed model analysis.** The expression variance for each gene was partitioned into the variance attributable to each variable using a linear mixed model implemented in variancePartition (v1.8.1) [39]. Categorical variables (i.e., cell type, donor, diagnosis, sex) were modeled as random effects and continuous variables were modeled as fixed effects. All remaining expression variation was termed residual. Each gene was considered separately and the results for all genes were aggregated afterwards.

**Enrichment analysis using publicly available datasets** Briefly, we first obtained a p-value for each gene to which at least one SNP was mapped (10kb upstream and 1.5kb downstream), using 1000 Genomes Project European reference panel (Phase 3) and accounting for correlations between SNPs based on the linkage disequilibrium. We then applied linear regression to test whether our DEGs were significantly associated with each of the disorders, including correction for gene size and SNP density.

**Inhibition of SGK1 and GSK3 and neurite imaging.**  Coverslips were fixed with 4% PFA/4% sucrose for 15 minutes, permeabilized using 0.25% Triton X-100 in PBS and blocked with 10% BSA for 30 minutes. Cells were stained using primary antibodies ms IgG1 FGF12 (NeuroMabs, catalog # 75-135, 1:200), Ck MAP2 (Invitrogen catalog #pa1-10005, 1:2000), and Rb βIII Tubulin (Abcam, catalog # ab18207; 1:2000) overnight at 4°C in 3% BSA. Isotype specific secondary antibodies ms IgG1 Alexa 647 (Life Technologies, 1:200), rb Alexa 488 (Life Technologies, 1:200) Ck Dylight 405 (Jackson ImmunoResearch Laboratories, catalog# 103-475-155, 1:200) in 3% BSA were applied for 2 hours. Cells were washed and mounted using ProLong Gold Antifade Mountant (ThermoFisher, catalog # P36930). Confocal images were acquired with a Zeiss LSM-880 with Airy scan confocal microscope with a 63X oil immersion objective (1.4 NA). Multi-track acquisition was done with excitation lines at 405 for Jackson Dylight 405, 488nm for Alexa 488, and 633nm for Alexa 647. Z-stacks were acquired every 0.43 mm with a frame size of 1024x1024 pixels and a scan speed of 7. Acquisition parameters, including photomultiplier gain and offset, were kept constant for each protein of interest. For analysis, Z-stacks were first summed, and then neurites were selected with the experimenter blinded to cell line and inhibitor condition. Two to three independent 250x250 pixel areas containing no somas were chosen for image and an ROI mask was created based off of the threshold of the βIII tubulin staining. Each coverslip was independently thresholded to ensure that the maximum number of neurites were captured. In total, 519 cells were analyzed (HC DMSO n=59, HC CHIR99021 n=56, HC CHIR99021+ GSK650394 n= 60, HC GSK650394 n= 60, SCZ DMSO n= 68, SCZ CHIR99021 n= 72, SCZ CHIR99021+ GSK650394 n= 72, SCZ GSK650394 n= 72). Average fluorescent intensity of βIII tubulin and FGF12 in neurites for each condition were analyzed using a Two-Way mixed model ANOVA with a Dunnett’s multiple comparisons test.

**Reverse transcription quantitative polymerase chain reaction** Total RNA samples (500 ng) were converted to cDNA using the High Capacity cDNA Reverse Transcription Kit (Thermo Fisher Scientific). Analysis was carried out in a QuantStudio™ 7 Flex Real-Time PCR System using Taqman Gene Expression Assays (Thermo Fisher Scientific) for specific targets (SGK1 – Hs00178612_m1, GAPDH – Hs99999905_m1). All samples were run in triplicate. Relative quantification (RQ) results were calculated using delta delta Ct and data were analyzed using GraphPad PRISM 6 software.
