# Supplementary material for: Convergent genomic and pharmacological evidence of PI3K/GSK3 signaling alterations in neurons from schizophrenia patients
Source: Neuropsychopharmacology. 2020 Dec 7;46(3):673–82. doi: 10.1038/s41386-020-00924-0 (PMC8027596; doi:10.1038/s41386-020-00924-0)
Supplement: Supplementary file 2 — Demographics and quality control [file 41386_2020_924_MOESM2_ESM.docx]

| Group | ID | Gender | Age* | iPSC Clone Passage | Mycoplasma Test | Karyotype | SSEA-4+, Oct-4+ | NPC batch for RNAseq | NPC batch for mechanistic studies | Neuron batch for RNAseq | Neuron batch for qPCR | Neuron batch for mechanistic studies |
| --- | --- | --- | --- | --- | --- | --- | --- | --- | --- | --- | --- | --- |
| HC | 353 | Female | 61 | 4 | Negative | Normal | Yes | 1 | N/A | 1 | N/A | N/A |
|  | 354 | Male | 41 | 5 | Negative | Normal | Yes | 1 | N/A | N/A | N/A | N/A |
|  | 357 | Female | 42 | 6 | Negative | Normal | Yes | 1 | N/A | 1 | N/A | N/A |
|  | 412 | Female | 31 | 5 | Negative | 5% of chromosome 1 aneuploidy | Yes | 1 | 2 | 1 | 2 | 2 |
|  | 417 | Female | 37 | 6 | Negative | Normal | Yes | 1 | 1 | 1 | 2 | 2 |
|  | 419 | Female | 38 | 6 | Negative | 5% of chromosome 1 aneuploidy | Yes | 1 | 2 | 1 | 2 | 2 |
| SZ | 352 | Male | 39 | 8 | Negative | Normal | Yes | 1 | 1 | 1 | 2 | 2 |
|  | 413 | Male | 34 | 8 | Negative | 5% of chromosome 1 aneuploidy | Yes | 1 | 2 | 1 | 2 | 2 |
|  | 414 | Male | 17 | 5 | Negative | Normal | Yes | 1 | N/A | N/A | N/A | N/A |
|  | 416 | Male | 35 | 8 | Negative | Normal | Yes | 1 | 2 | 1 | N/A | N/A |
|  | 418 | Male | 40 | 4 | Negative | 5% of chromosome 1 aneuploidy | Yes | 1 | 2 | 1 | 2 | 2 |
|  | 486 | Male | 41 | 5 | Negative | Normal | Yes | 1 | N/A | 1 | N/A | N/A |
|  | 624 | Female | 44 | 7 | Negative | Normal | Yes | 1 | N/A | 1 | N/A | N/A |

*Age at time of blood donation. Only one iPSC clone from each subject was used to generate the hiPSC-NPC. For the cell lines with two batches of hiPSC-NPC, the hiPSC clone used for the second batch was the same one used on the first batch, with only one or two passages higher.
