# Supplementary material for: Convergent genomic and pharmacological evidence of PI3K/GSK3 signaling alterations in neurons from schizophrenia patients
Source: Neuropsychopharmacology. 2020 Dec 7;46(3):673–82. doi: 10.1038/s41386-020-00924-0 (PMC8027596; doi:10.1038/s41386-020-00924-0)
Supplement: Supplementary file 4 — Concordance of DEGs with SCZ gene expression studies [file 41386_2020_924_MOESM4_ESM.docx]

**Supplementary Table 3.** Concordant differential gene expression between the current study and studies of dorsolateral prefrontal cortex gene expression in schizophrenia.

|  |  | **Stertz et al** | | | | **Fromer et al 2016 (Common Mind)^46^** | | | |
| --- | --- | --- | --- | --- | --- | --- | --- | --- | --- |
|  | **SYMBOL** | **logFC** | **t** | **P** | **Adj.P** | **logFC** | **t** | **P** | **Adj.P** |
| ENSG00000002587 | HS3ST1 | 1.264994 | -3.363 | 0.0007 | 0.04303 | -1.01E-01 | -3.345 | 8.77E-04 | 3.46E-02 |
| ENSG00000017427 | IGF1 | 0.966514 | -3.449 | 0.00056 | 0.03694 | 1.70E-01 | 3.762 | 1.86E-04 | 1.63E-02 |
| ENSG00000084636 | COL16A1 | 1.584271 | -5.226 | 1.73E-0 | 0.00026 | -1.29E-01 | -3.095 | 2.06E-03 | 4.96E-02 |
| ENSG00000104327 | CALB1 | 1.267835 | -3.370 | 0.0007 | 0.04249 | 2.12E-01 | 4.406 | 1.26E-05 | 4.39E-03 |
| ENSG00000108256 | NUFIP2 | -0.88044 | 3.7919 | 0.00014 | 0.01857 | 8.76E-02 | 3.587 | 3.63E-04 | 2.33E-02 |
| ENSG00000114439 | BBX | -0.81163 | 3.2809 | 0.00103 | 0.04894 | 8.19E-02 | 3.297 | 1.04E-03 | 3.81E-02 |
| ENSG00000129009 | ISLR | 1.760063 | -5.239 | 1.61E-07 | 0.00026 | -1.25E-01 | -3.127 | 1.86E-03 | 4.81E-02 |
| ENSG00000132164 | SLC6A11 | 1.423619 | -3.916 | 8.97E-05 | 0.01399 | -1.29E-01 | -3.588 | 3.62E-04 | 2.33E-02 |
| ENSG00000132622 | HSPA12B | 1.300649 | -3.519 | 0.000432 | 0.03296 | -1.91E-01 | -3.852 | 1.30E-04 | 1.42E-02 |
| ENSG00000133460 | SLC2A11 | 0.938860 | -3.494 | 0.000474 | 0.03415 | 0.03415826 | -3.446 | 6.11E-04 | 2.93E-02 |
| ENSG00000135744 | AGT | 1.498958 | -4.313 | 1.60E-05 | 0.00466 | -1.50E-01 | -3.212 | 1.39E-03 | 4.35E-02 |
| ENSG00000152242 | C18orf25 | -0.69298 | 3.9594 | 7.51E-05 | 0.01268 | 5.93E-02 | 3.244 | 1.25E-03 | 4.12E-02 |
| ENSG00000152409 | JMY | -0.60185 | 3.4233 | 0.000618 | 0.03905 | 6.85E-02 | 3.149 | 1.72E-03 | 4.74E-02 |
| ENSG00000158615 | PPP1R15B | -0.81767 | 3.3523 | 0.000801 | 0.04312 | 7.14E-02 | 3.336 | 9.04E-04 | 3.50E-02 |
| ENSG00000158711 | ELK4 | -1.15403 | 3.5737 | 0.000352 | 0.02983 | 8.93E-02 | 3.142 | 1.77E-03 | 4.76E-02 |
| ENSG00000164463 | CREBRF | -1.19996 | 4.1662 | 3.10E-05 | 0.0068 | 7.21E-02 | 3.412 | 6.91E-04 | 3.08E-02 |
| ENSG00000165092 | ALDH1A1 | 1.261812 | -3.876 | 0.000106 | 0.01552 | -2.74E-01 | -5.609 | 3.17E-08 | 1.73E-04 |
| ENSG00000167114 | SLC27A4 | 0.696408 | -3.481 | 0.000499 | 0.03503 | -9.09E-02 | -3.253 | 1.21E-03 | 4.06E-02 |
| ENSG00000172795 | DCP2 | -0.93980 | 4.2105 | 2.55E-05 | 0.00610 | 1.00E-01 | 3.484 | 5.31E-04 | 2.76E-02 |
| ENSG00000198797 | BRINP2 | 1.568597 | -4.333 | 1.47E-05 | 0.00454 | -6.97E-02 | -3.112 | 1.95E-03 | 4.90E-02 |

|  |  | **Stertz et al** | | | | **Bowen et al 2019 (NIMH)^47^** | |
| --- | --- | --- | --- | --- | --- | --- | --- |
|  | **SYMBOL** | **logFC** | **t** | **P** | **Adj.P** | **Beta** | **P** |
| ENSG00000103316 | CRYM | 1.775986 | -4.83609 | 1.32E-06 | 0.000891 | -0.2266 | 0.029963 |
| ENSG00000117519 | CNN3 | -0.72911 | 3.799152 | 0.000145 | 0.018178 | 0.2516 | 1.78E-06 |
| ENSG00000136636 | KCTD3 | -0.70926 | 3.508514 | 0.000451 | 0.033416 | 0.1307 | 0.00638 |
| ENSG00000137145 | DENND4C | -0.7924 | 3.343932 | 0.000826 | 0.043811 | 0.097 | 0.024905 |
| ENSG00000164463 | CREBRF | -1.19996 | 4.166294 | 3.10E-05 | 0.006803 | 0.097 | 0.024905 |
| ENSG00000179454 | KLHL28 | -0.87085 | 3.830041 | 0.000128 | 0.016926 | 0.1216 | 0.049015 |
