# Supplementary figures and images for: Convergent genomic and pharmacological evidence of PI3K/GSK3 signaling alterations in neurons from schizophrenia patients
Source: Neuropsychopharmacology. 2020 Dec 7;46(3):673–82. doi: 10.1038/s41386-020-00924-0 (PMC8027596; doi:10.1038/s41386-020-00924-0)

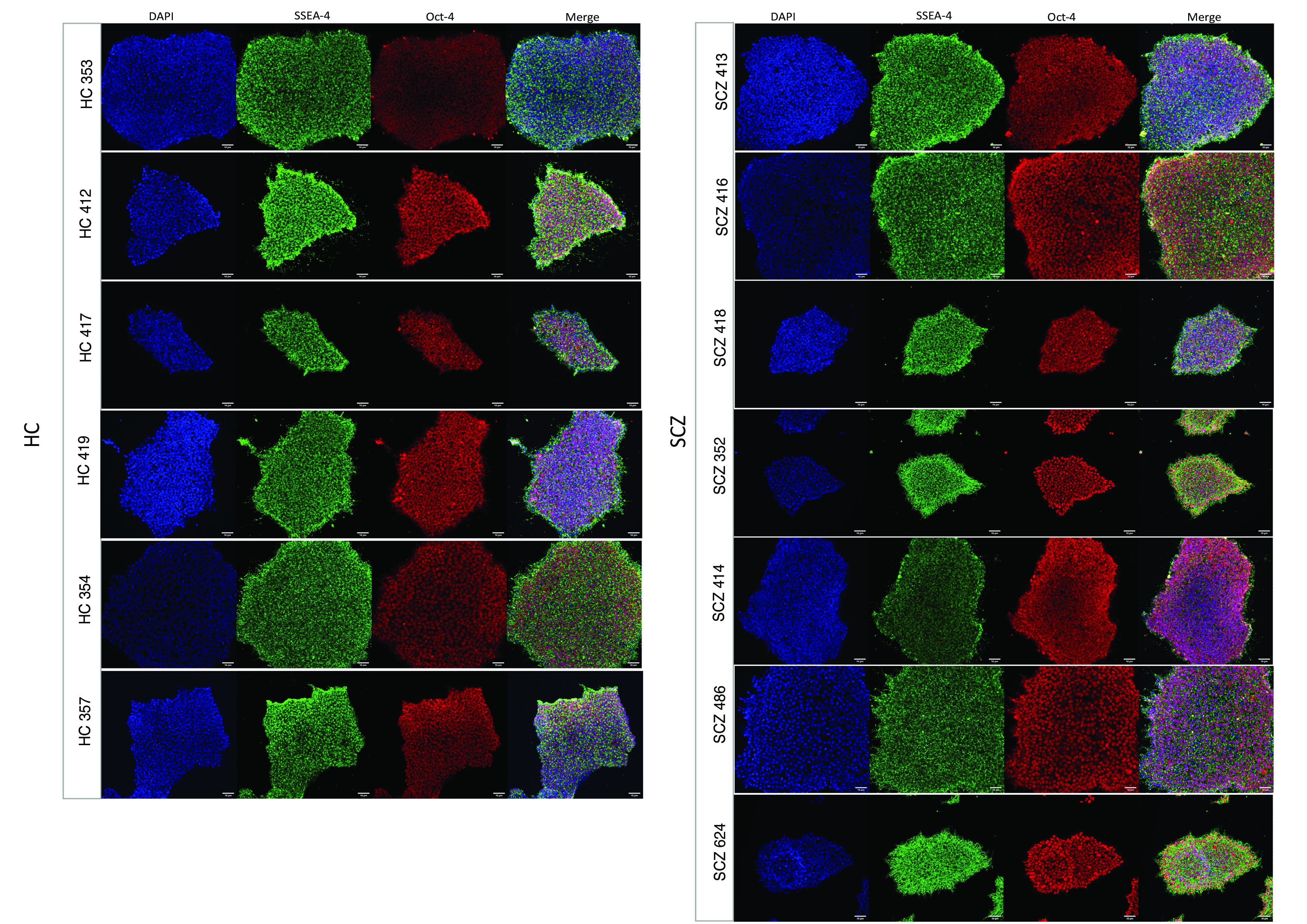

Supplement: Supplementary file 5 — Characterization of hiPSCs generated from LCLs [file 41386_2020_924_MOESM5_ESM.tif]

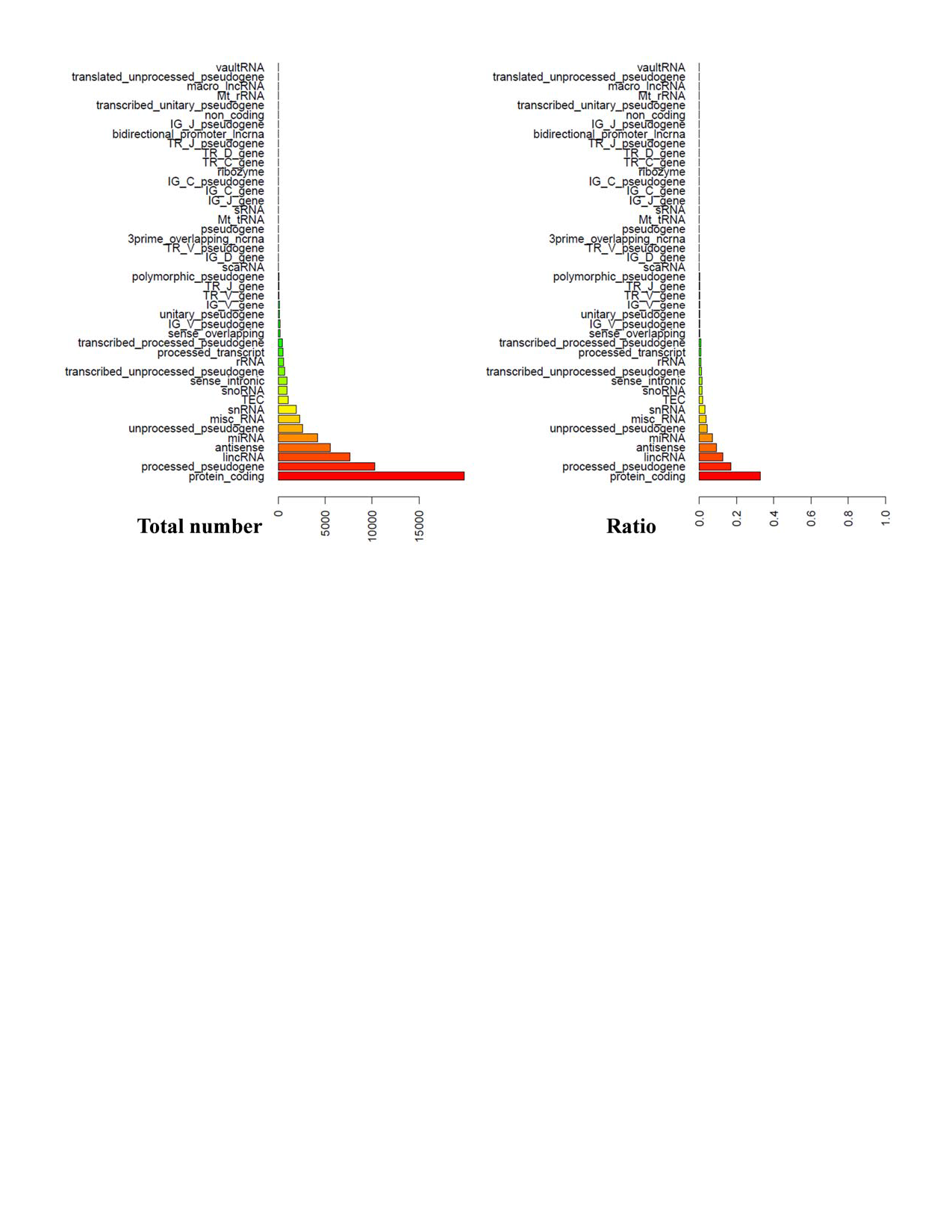

Supplement: Supplementary file 6 — Gene feature statistics [file 41386_2020_924_MOESM6_ESM.tif]

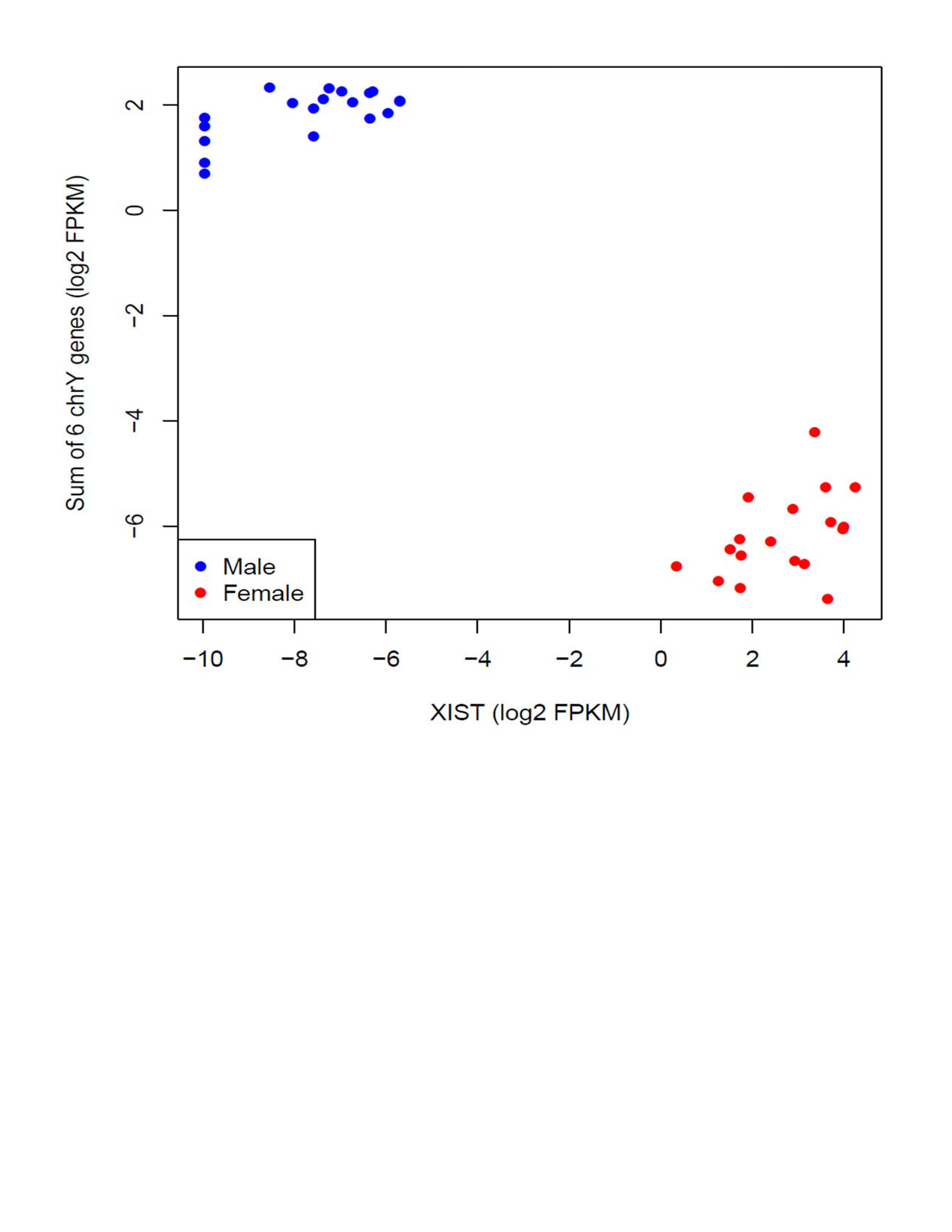

Supplement: Supplementary file 7 — Expression pattern of sex chromosome genes [file 41386_2020_924_MOESM7_ESM.tif]

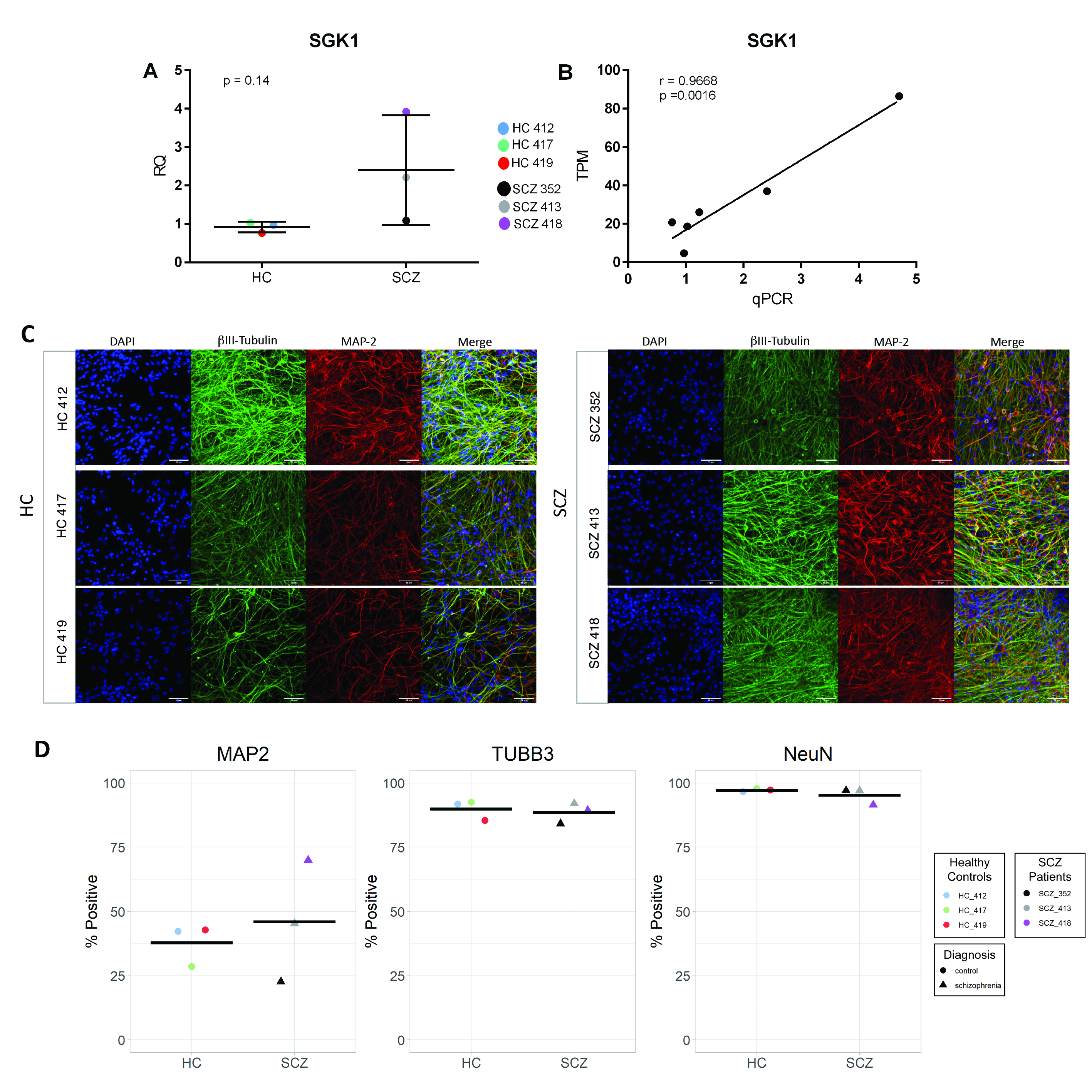

Supplement: Supplementary file 9 — Characterization of neurons in functional studies [file 41386_2020_924_MOESM9_ESM.tif]
